# Repellency Effect of Essential Oils of some Native Plants and Synthetic Repellents against Human Flea, *Pulex irritans* (Siphonaptera: Pulicidae)

**Published:** 2017-03-14

**Authors:** Mohammad Bagher Ghavami, Fahimeh Poorrastgoo, Behrooz Taghiloo, Jamshid Mohammadi

**Affiliations:** 1 Department of Medical Entomology and Vector Control, Faculty of Medicine, Zanjan University of Medical Sciences, Zanjan, Iran; 2 Zanjan Health Center, Zanjan University of Medical Sciences, Zanjan, Iran

**Keywords:** *Pulex irritans*, *Ziziphora tenuiore*, *Myrtus communis*, DEET, Repellents

## Abstract

**Background:**

Fleas are important vectors of human and animal disease, and control measures for protection against their bites and flea-borne diseases are necessary.

**Methods:**

The essential oils (EOs) of four native medicinal plants, *Ziziphora tenuiore*, *Myrtus communis*, *Achillea wilhelmsii* and *Mentha piperita* were isolated by hydrodistillation technique and analyzed by GC-MC. The repellent activity of EOs and synthetic compounds, DEET and permethrin, were assayed on human subjects against field collected fleas. The effective doses of 50% and 90% of EOs and synthetic compounds were estimated by probit analysis of dose and response regression line.

**Results:**

Analysis of EOs revealed about 19 major components. All oils were found to be more repellent (ED_50_ range= 208–955μg cm^−2^) than DEET and permethrin (ED_50_ range= 27–182 × 10^3^μg cm^−2^). Thyme and myrtle oils showed high repellent activities and among the total detected terpenes, thymol (36.26%) and α-pinene (32.5%) were the major components of those oils respectively.

**Conclusion:**

Low repellent potency of DEET and permethrin against fleas might be related to flea olfactory system and further molecular and electrophysiological studies are required to conceive new ideas for the discovery and development of the next generation of repellents. Based on high repellent activity of thyme and myrtle essential oils against *Pulex irritans* further studies should be staged to develop their appropriate effective formulations. Likewise, field trials should be carried out to evaluate the operational feasibility and dermal toxicity over a long period.

## Introduction

The human flea, *Pulex irritans* Linnaeus, is the most invasive species worldwide with great medical importance and intolerable nuisance. The control of this vector has received considerable attention since ancient times. The most effective measure to control human flea lies in adult stage using adulticides (Service 2012). Although application of synthetic insecticides can be effective, their intensive use has caused concerns regarding their impact on human and environmental health leading to development of resistance in the flea populations like other vectors (Hemingway and Ranson 2000). In addition to adulticides, personal protection including the use of repellents can decrease contact between human and fleas. N, N-Diethyl-3-methylbenzamide (DEET) has been considered as one of the most effective synthetic repellents against various groups of vectors and still remains as “the gold standard” among currently available insect repellents (Swal et al. 2014, Debboun et al. 2014). However, there has been no report concerning the repellency of DEET on human flea. In addition, there have been case reports of DEET toxicity especially among children and the elderly (Qui 1998, Sudakin 2003). Moreover, genotoxic effects of this compound have been found previously (Manikhan et al. 2012). In addition to DEET, permethrin, a synthetic pyrethroid, is characterized by a high level of potency against a wide range of vectors, rapid reactivity, excellent photostability, resistance to weathering, low mammalian toxicity (Yu 2014) and high potency in tick and insect bite prevention (Faulde et al. 2003, 2006). However, skin irritation, including itching, swelling, redness, mild burning and stinging may occur after treatment with permethrin. Moreover, arthropods show differential responses to these products, indicating the possibility of adaptation and emerging resistance or insensitivity (Klun et al. 2004, Stanczyk et al. 2010, Pellegrino et al. 2011, Sfara et al. 2011, Del Fabbro and Nazzi 2013). Thus, there is a growing demand to evaluate natural repellents to develop suitable agents for prophylactic treatment in integrated pest management.

Plant essential oils (EOs) specially their monoterpen components; exhibit a variety of biological activities against a wide spectrum of insect pests. They can adversely affect the growth and reproduction rate, behavior trait of insect pests and act as contact insecticide, fumigant, repellent and antifeedent agent. These compounds have a low risk profile on the environment, mammals, and humans and might comprise natural alternatives for conventional insecticides (Bakkali et al. 2008, Koul et al. 2008).

Fresh and dried shoots of myrtle, mint, thyme, yarrow and their EOs and extracts are widely used in cosmetic, food, gum, toothpaste and pharmaceutical industries. These plants have also been commonly used in folk medicine for treatment of colds, pains and as spices and herbal tea as well (Naghibi et al. 2005, Bakkali et al. 2008, Saeidnia et al. 2011). Moreover, these plants proved to be toxic to different species of insects and ticks (Maia and Moore 2010, George et al. 2008, 2009, 2014, Pitarokili et al. 2011, Yahoobi-Ershadi et al. 2011, Tavassoli et al. 2011, Koc et al. 2012, Lupi et al. 2013).

For human flea, however, little work has been done on EOs repellent activity. Moreover, despite the great need in human health area for fleas prophylaxes by natural products, the current knowledge concerning repellent action of EOs on human flea is very limited. Therefore, in the present study the chemical properties and components of some EOs were evaluated. Additionally a comparative study was conducted for detection of flea repellent activity of these Eos.

## Materials and Methods

### Plant materials

The shoots of native medicinal plants, thyme (*Ziziphora tenuiore*), mint (*Mentha piperita*), yarrow (*Achillea wilhelmsii*) and myrtle (*Myrtus communis*) were collected from different areas of Zanjan and Fars Provinces during May–Jun 2013 and 2014. Collected herbs were dried in shade for 8 days at room temperature until they became brittle. Air-dried samples were powdered in electric vegetable chopper and subjected to hydro distillation in a Clevenger type apparatus for about 3h. The extracted oils were dried over anhydrous sodium sulfate and refrigerated at 4 °C in dark glass bottles until the experiment.

### Gas Chromatography and GC mass analysis

The gas chromatography (GC) analysis was carried out with an HP Series II 7890N instrument equipped with flame ionization detector and HP-5-MS capillary column (30m× 0.25mm, 0.25μm film thickness). Injector and detector temperatures were set at 260 °C and 270 °C respectively. Thermal program of GC oven started with 60 °C for 4min, then rose to 60–225 °C at a rate of 3oc/min. Helium was the carrier gas at a flow rate of 1ml/min. Diluted samples (1:50 in diethyl ether) of 1μl were injected manually in the split less mode.

Quantity analysis of EOs components was performed using the same condition with GC HP Series II equipped with HP GC 5975C mass selective detector in the electron impact mode 70eV. The oil components were identified by comparison of the retention time with those of authentic samples in mass spectra obtained from literature and the library of the GC-MS system.

### Human subjects

The study was conducted on 6 volunteers (four males and two females) who were aged between 28 to 50 years old. They were nonsmokers, non alcoholics and non-allergic to insect bites or herbal oils. They also had no contact with lotions, perfumes, coils or perfumed soaps on the days of assay. All volunteers signed an formal consent form after having received a full explanation of the test objectives, procedures and foreseeable risk to subject. The research protocol was approved by the ethics committee of Zanjan University of Medical Sciences.

### Flea collection

Alive samples of fleas were collected by human baits from animal shelters of Khodabandeh and Mahneshan districts of Zanjan Province from September 2013 to October 2014. Volunteers in white overalls and white ladies stockings, walked around the houses for 3–5 minutes and attracted fleas were suctioned by electrical aspirator and transferred to laboratory in 2 liters Erlenmeyer flasks.

### Preparation of solutions

Dilutions of each essential oil and DEET (Aldrich USA, D100951-500G, N, N-Diethyl-3-methylbenzamide, lot# MKBH0428V, P Code: 1001075635, Density: 0.97g/cm^3^) with grade reagent ethanol (ET) were made to stock in *V/V* at 85% concentration. From which five serial dilutions (42.75%, 21.37%, 10.68% and 5.34% concentrations) were prepared. Also a 20% permethrin (United Phosphorus Ltd, Delhi India, with 96% active ingredient, 42:58 cis: trans ratio) solution was prepared by mixing 2.1g of permethrin with 10ml of grade reagent acetone and 5 serial dilutions (in acetone) were created.

### Repellency tests

The bioassay was performed by the standard protocols for testing mosquito and tick repellents (Dautel 2004, Bissinger 2010, Maia and Moore 2011, Krober et al. 2013, WHO 2013, Debboun 2014). About 60 unfed fleas were kept in laboratory condition (25±2 °C and 60% RH) and transferred to 20″×6″ clear cylinder glass vase. For testing, volunteers used the left arm for treatment and the right for control. Five hundred μl of test materials were applied to the treated area of left hand (from elbow to wrist, area approximately 500cm^2^) and allowed to dry for 5 minutes. The same area of right arm was applied with five hundred μl of ET. After drying of treated area, both arms were covered with white ladies stockings and volunteers kept their hands (left as treatment and right as control) in glass jars about 3 minutes. The control hand was exposed before the treatment. Samples of fleas from different parts of arms (hand and wrist to elbow) were collected and recorded separately. These samples were preserved in ET and observed under stereomicroscopy at 30×. Samples of *Pulex irritans* were recorded and other species of fleas were excluded from study. The percentage of repellency in each hand was calculated by the following formula: (Tc–Tb)/(Tc+Tb)× 100, Where Tc= the number of fleas in hand of arm and Tb is the numbers in the area between elbow to wrist. The percentage of repellency in treatment groups was corrected with control group by Abbot’s formula.

The data were subjected to probit analysis and the median effective dose (ED_50_) and 95 % effective dose (ED_90_) with 95 confidence interval exposed in microgram of repellent per square of centimeter of skin area were estimated. Significant differences between effective dose values were determined by comparing the confidence intervals of the values.

## Results

Water distillation of the dried shoots of the examined plants furnished 0.8–1.2% (W/W) of white to yellowish essential oils with strong pleasant odors were identified. Forty seven constituents were determined in the thyme essential oil representing 97.98% of total content. The main constituents were found to be thymol (36.26%), geraniol (11.61%), carvacrol (4.96%) and 1,8 cineol (3.26%) (Table 1).

**Table 1.** Relative percentage of major components of essential oils of each plant extract

**No**	**Compound name**	**LRI (Min)**	***Myrtus communis***	***Achillea wilhelmsii***	***Mentha piperita***	***Ziziphora tenuiore***
**1**	α-Pinene	6.37	32.5	2.59	0.73	0.71
**2**	Limonene	10.24	15.5	0	0	0
**3**	1,8 Cineol	10.36	24.15	3.86	8.6	3.16
**4**	γ-Tepinene	11.55	0	0.33	0.11	3.33
**4**	Artemisia alcohol	12.86	0	5.39	0	0
**5**	Linalooll	13.42	10.6	3.2	0.88	2.26
**6**	Dimethyhepta	14.67	0	10.15	0	0
**7**	Camphor	15.29	0	19	0.48	0.69
**8**	Borneol	16.38	0	7.30	0.35	3.26
**9**	Menthen	17.55	0	4.56	26.66	2.41
**11**	α-Terpinol	17.7	0	1.17	0.44	2.36
**12**	Linalyl acetate	21.14	5.5	0	0.25	0
**13**	Geraniol	21.45	0	0	0	11.61
**14**	Thymol	23.35	0	0.8	0.22	36.26
**15**	Carvacrol	23.57	0	0.26	0	7.98
**16**	Piperiton	24.99	0	0	19.18	0
**17**	Piperiton oxide	26.11	0	0	19.07	0
**18**	Geranyl acetate	26.68	0	0	0.40	4.72
**19**	Trans caryophylen	27.83	0	0	3.29	0

**Total**			88.25	57.76	76.8	78.75
**Total Compound (No)**			20	80	58	47
**Total percentage**			99.15	87.43	98.26	97.98

LRI*= Linear regression indices on HP-5 MS column relative to C_9_–C_23_ n alkanes, identification based on comparison of mass spectra obtained from the library of the GC-MS system and from literature.

A total of twenty compounds were identified in the oil of myrtle, representing around 99.15% of the oil totally. Alpha pinene (32.5%), 1,8 cineol (24.15), limonene (15.5%) linalool (10.6%) and linalyl acetate (5.5%) were the major constituents in the essential oil of *Myrtus communis* (Table 1)

According to total 87.43% of the *Achillea wilhelmsii* oil, eighty constituents were identified in the essential oil of this plant. The main components were camphen (19%), dimethyl heptatriene (10.15%), borneol (7.3%), artemisia alcohol (5.3%), menthen (4.56%) and 1,8 cineol (3.86%).

There were fifty eight compounds in the oil of *Mentha pipertta*, comprising 98.26% of the total weight. Menthen (26.66%), piperiton (19.18%), piperiton oxide (19.07%), 1,8 cineol (8.5%) and trans caryophylen (3.29%) were the major compounds of essential oil of this plant (Table 1).

During the study, 4529 samples of fleas were collected by human-bait catch. Human flea was the commonest species and 94.3% of samples were identified as *Pulex irritans.* The frequencies of other fleas were 3.8% and 1.9 % for *Ctenocephalidescanis* and *C. felis* respectively. The bioassay tests were carried out with 4271 samples of alive human flea.

All tested essential oils in five concentrations showed differences in repellency with chemical compounds. The thyme essential oil provided highest repellency followed by myrtle, yarrow and mint essential oils (Table 2).

**Table 2.** Repellent activity (in percentage) of some essential oils and synthetic compounds against *Pulex irritans* on human volunteers

**Dosage (μ g/cm^2)^**	**Study groups**					
	***Ziziphora tenuiore***	***Myrtus communis***	***Achillea wilhelmsii***	***Mentha piperita***	**Permethrin**	**DEET**
**50**	3.2	3	3.1	3	2	2
**100**	17.7	17.5	12.5	15	7.2	5.2
**200**	48	36	22	20	8.2	6.2
**400**	74	63	46	32.5	9.3	7.3
**800**	87.5	80	72	48.4	12.4	10.4
**1600**	98	96	87	69	18.5	14.6

The EDs_50_ (with 95% confidence intervals) of thyme and myrtle essential oils were 229 (208–257) and 295 (269–338)μg/cm^2^ respectively. In these plants the ED_90_ (with 95% confidence intervals) of essential oils were 776 (660–933) and 1172 (988–1412) μg/cm^2^ respectively (Table 3).

**Table 3.** Parameters of probit analysis on botanical and chemical compounds and their repellency against *Pulex irritans* on human volunteers

**Compound**	**a**	**b**	**ED_50_ (95 % CE) (μ g/cm^2^)**	**ED_90_ (95 % CE) (μ g/cm^2^)**	**n**	***X*^2^**	**p**
***Ziziphora tenuiore***	−5.78	2.44	229 (208–257)	776 (660–933)	828	101.7	<0.01
***Myrtus communis***	−5.36	2.16	295 (269–338)	1172 (988– 1412)	895	99.6	<0.01
***Achillea wilhelmsii***	−5.0	1.88	457 (371–630)	2217 (1399–4673)	169	102.1	<0.01
***Mentha piperita***	−4.32	1.49	776 (660–955)	5717 (3890–9120)	822	68.7	<0.01
**Permethrin**	−2.3	0.51	26.9 × 10^3^	7.76 × 10^6^	362	15.0	<0.01
**DEET**	−2.74	0.50	182 × 10^3^	51 × 10^6^	710	10.8	0.03

Yarrow essential oil showed moderate repellency against human flea. In this plant the ED_50_ and EDs_90_ (with 95% confidence intervals) of essential oil were 457 (371–630) and 2217 (1399–4673)μg/cm^2^ respectively. Mint essential oil provided slight repellency. The ED_50_ and ED_90_ (with 95% confidence intervals) of mint essential oil were 776 (660–855) and 5717 (3890–9120)μg/cm^2^ respectively.

Permethrin and DEET compounds showed low repellency in comparison with botanical essential oils. The ED_50_ of permethrin and DEET were estimated 7760 and 51000μg/cm^2^ respectively (Table 3).

**Fig. 1.**
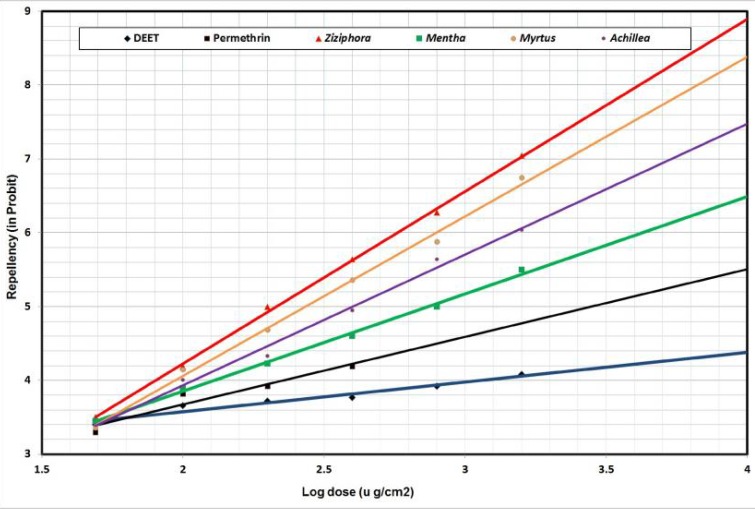
Dose response lines for botanical and chemical repellent activity against *Pulex irritans* on human subjects.

The related dose response lines and regression equations for essential oils and synthetic compounds are represented in Table 3 and Fig. 1.

## Discussion

Evaluation of botanical compounds is essential to identify effective repellents for vector control programming and protection of populations against vector nuisance and vector borne diseases.

The insecticidal and repellent activities of various herbal derivatives have been demonstrated in many studies (Neiro et al. 2010, Bisinger and Roe 2010, Lupi et al. 2013).

However, only a few reports present the repellency of plant products against other groups of nuisance vectors especially fleas (George et al. 2009). In our investigation among examined plants, the essential oil of three plants, thyme, myrtle and yarrow, showed excellent to good repellency against all tested fleas. The oils of these plants were rich in volatile terpenes, thymol, α–pinene, 1, 8 cineol, camphor, limonene, linalool, and geraniol. In spite of effective repellency of these compounds, the major components of *Mentha piperita*, menthen and piperiton, showed low efficacy against fleas.

Findings of previous studies have proved that, thymol and α–pinene are the most toxic compounds (Trabouli et al. 2002) and have effective repellent activity against mosquitoes and ticks (Pandy et al. 2009, Jordan et al. 2012, Meng et al. 2015). High repellency of thyme and myrtle oils in our study might be associated with these components. Nevertheless, further laboratory and field studies are required to prove repellent efficacy of these compounds.

Although the amounts of limonene, geraniol, linalool and camphor in above-mentioned essential oils were high in our study, they showed low potency of repellent activity against fleas. Furthermore, 1, 8 cineol which had low repellency against insects, showed different biological activities against mosquitoes and ticks in previous studies (El-Seedi et al. 2012).

In contrast to recent studies synthetic compounds, DEET and permethrin, which have shown effective repellency against mosquitoes, could not repel human flea. Low efficacy of these compounds on *Pulex irritans* compared to other vectors might be related to their difference in mode of action (Dikens and Bohbot 2013, Daniel et al. 2014), molecular characteristics of odorant binding proteins (Bohbot and Dikens 2012) and odorant receptors of fleas (Ditzen et al. 2008). This study encourages further electrophysiological and molecular studies on human flea to conceive new ideas for the discovery and development of the next generation of repellents.

Thyme and myrtle oils showed high repellent activity among the tested oils against human flea on human subjects. The repellent effect of *Ziziphora tenuiore* essential oil showed the highest repellency (lowest ED_50_ value, 208μg/cm^2^ and ED_90_ 910μg/cm^2^). According to our literature review, there is no published paper available on the repellent activity of *Ziziphora tenuiore* essential oil. Majority of published documents were in favor of thyme essential oil repellency against mosquitoes and only a few of them were conducted against ticks (Zhu et al. 2006, Park et al. 2012). In previous studies the lowest value of ED_50_ seen in laboratory study of *Origanium majorans*, a species of thyme group, against nymphs of *Ixodesricinus* was 15μg/cm^2^ (Zhu et al. 2006). Thyme oil also showed highest repellency against lab-bred *Aedes aegypti* (ED_50_= 23μg/cm^2^) and *Culex pipiens* (ED_50_= 468μg/cm^2^).

Our ED_50_ and ED_90_ estimated values for myrtle oil on human subject were 290 and 1172μg/cm^2^ respectively. In the laboratory study of Tavassoli et al. (2011) the ED_50_ and ED_90_ values for myrtle essential oil against lab-bred *Anopheles stephensi* were calculated as 110 and 540μg/cm^2^ respectively. Moreover, in a laboratory study on animal model, using K and D apparatus against lab reared *Phlebotomus papatasi*, Yaghoobi-Ershadi et al. (2006) reported the ED_50_ and ED_90_ values of myrtle oil 114 and 671μg/cm^2^ respectively. Interestingly, in our study the ED_50_ and ED_90_ values of myrtle oil are very close to these studies.

Based on the repellent result against *Pulex irritans* we recommend *Ziziphora tenuiore* and *Myrtus communis* essential oils for further studies to develop appropriate effective formulations. Field trials should be carried out to evaluate the operational feasibility and dermal toxicity over a long period. It is important to determine whether widespread application of these repellents would produce an overall reduction of vector biting.

## Conclusion

Based on our findings the two essential oils, thyme and myrtle, are preferable in terms of repellency effect against human flea. Further electrophysiological and molecular studies are recommended to identify the mode of action of these oils and nature of flea olfactory system in order to develop new generation of repellents.

Measures to determine the appropriate formulation of recommended repellents and comprehensive field studies to evaluate their potency in bite reduction and possible adverse effects are necessary.
